# Supplementary material for: A dual fluorescent-Raman bioorthogonal probe for specific biosynthetic labeling of intracellular gangliosides
Source: Commun Chem. 2025 Oct 3;8:293. doi: 10.1038/s42004-025-01685-x (PMC12494992; doi:10.1038/s42004-025-01685-x)
Supplement: Supplementary file 2 — Supplementary Information [file 42004_2025_1685_MOESM2_ESM.pdf]

# A Dual Fluorescent-Raman Bioorthogonal Probe for Specific Biosynthetic Labeling of Intracellular Gangliosides

Mana Mohan Mukherjee[1], Matthew D. Watson[2], Devin Biesbrock[1], Lara K. Abramowitz[1], Steven K. Drake[3], Jennifer C. Lee[2], John A. Hanover[1]*

[1] Laboratory of Cell and Molecular Biology, NIDDK, NIH, Bethesda, MD, USA
[2] Laboratory of Protein Conformation and Dynamics, NHLBI, NIH, Bethesda, MD, USA
[3] Critical Care Medicine Department, Clinical Center, NIH, Bethesda, MD 20892, USA

Corresponding Author:
John A. Hanover
Laboratory of Cell and Molecular Biology
National Institute of Diabetes and Digestive and Kidney Diseases
National Institutes of Health
Bethesda, MD 20892
301-496-0943
johnh@bdg8.niddk.nih.gov

# Supplementary Figures

# Table of content for supplementary figures files


**Supplementary Figure 1.** NMR spectra of the MM-JH-2 β-isomer ........ Page 3

**Supplementary Figure 2.** NMR spectra of the MM-JH-2 α-isomer. ........ Page 4

**Supplementary Figure 3**. Concentration dependent incorporation of MM-JH-2 into SH-SY5Y neuroblastoma cells ........ Page 5

**Supplementary Figure 4.** Concentration dependent incorporation of MM-JH-2 into LA-N-2 neuroblastoma cells ........ Page 6

**Supplementary Figure 5.** Concentration dependent incorporation of MM-JH-2 into HEK293T epithelial cells ........ Page 7

**Supplementary Figure 6**. Concentration dependent incorporation of MM-JH-2 into NIH 3T3 epithelial cells ........ Page 8

**Supplementary Figure 7.** Concentration dependent incorporation of MM-JH-2 into GnT-I mutated LecCHO epithelial cells ........ Page 9

**Supplementary Figure 8**. Concentration dependent incorporation of MM-JH-2 into AML12 mouse hepatocytic cells ........ Page 10

**Supplementary Figure 9.** MM-JH-2 selectively labels lipids in cultured HeLa cells ........ Page 11

**Supplementary Figure 10.** Glucosylceramide synthase inhibitor Genz-123346 reduces MM-JH-2 labeling in HeLa cells ........ Page 12

**Supplementary Figure 11.** ST3GAL5 siRNA knockdown reduces MM-JH-2 labeling in SH-SY5Y cells ........ Page 13

**Supplementary Figure 12.** MALDI mass spectrometric analysis identifies MM-JH-2 modified GM3 gangliosides in lipid extracts ... Page 14

**Supplementary Figure 13.** MM-JH-2 localizes to lysosomes and late endosomes for catabolism ........ Page 15

**Supplementary Figure 14.** MM-JH-2 endocytosis depends on both dynamin and caveolin ........ Page 16

**Supplementary Figure 15.** Confocal imaging of HeLa cells treated with MM-JH-2 and $Pr_4$ManNAlk ........ Page 17

**Supplementary Figure 16.** Raman spectral imaging of HeLa cells treated with MM-JH-2 ........ Page 18

**Supplementary Figure 17.** MM-JH-2 selectively eradicates cancer cells over nonmalignant cells ........ Page 19

**Supplementary Figure 18.** MM-JH-2 can selectively label B cells over T cells in a coculture of splenocytes ........ Page 20

**Supplementary Figure 19.** Full uncropped western blots showing *ST3GAL5* siRNA knockdown reduces *ST3GAL5* labeling ........ Page 21

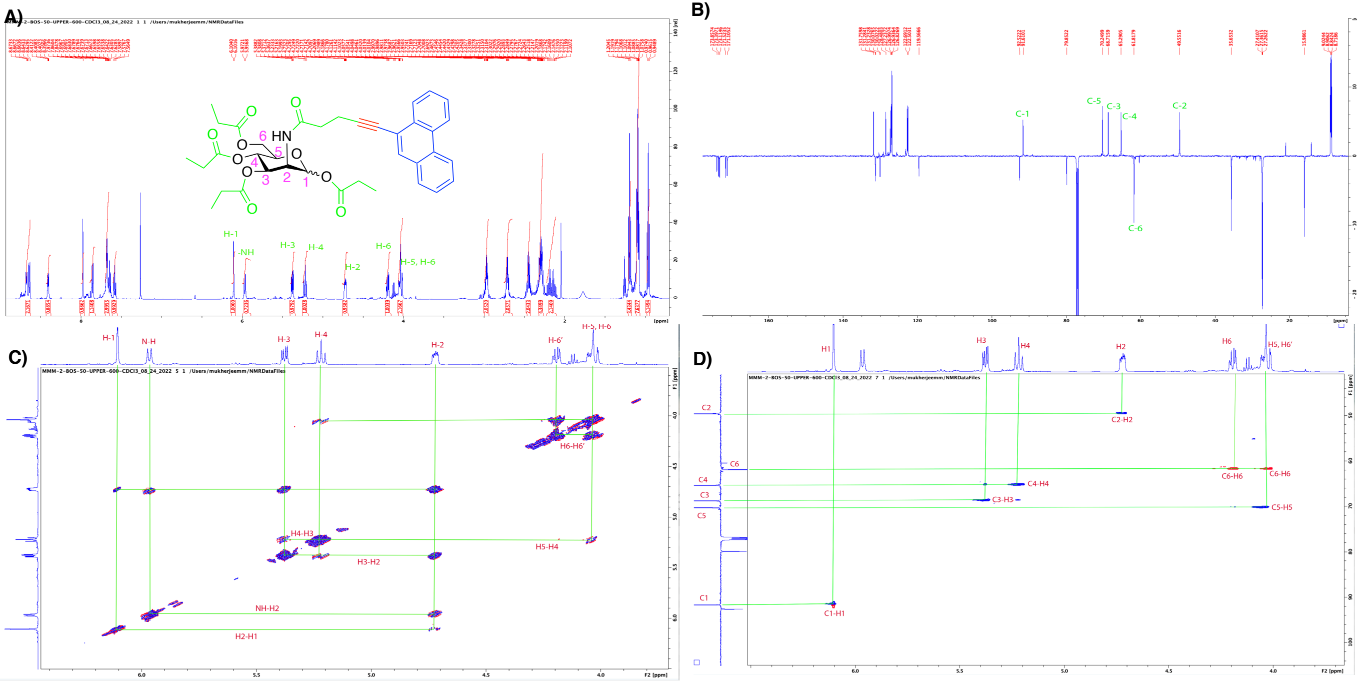


**Supplementary Figure 1.** NMR spectra of the MM-JH-2 β-isomer; (A) $^{1}$H NMR (600 MHz); (B) $^{13}$C NMR (150 MHz); (C) $^{1}$H-$^{1}$H COSY; (D) $^{1}$H-$^{13}$C HSQC.

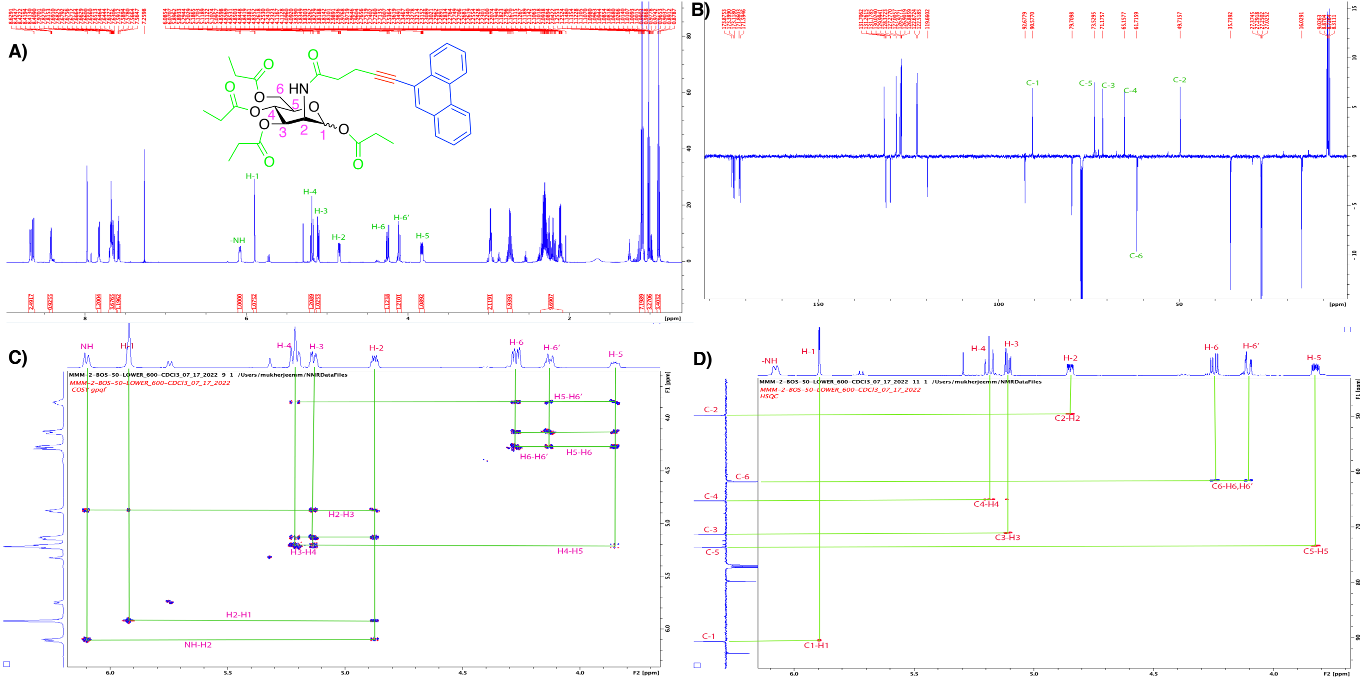


**Supplementary Figure 2.** NMR spectra of the MM-JH-2 α-isomer; (A) $^{1}H$ NMR (600 MHz); (B) $^{13}C$ NMR (150 MHz); (C) $^{1}H$-$^{1}H$ COSY; (D) $^{1}H$-$^{13}C$ HSQC.

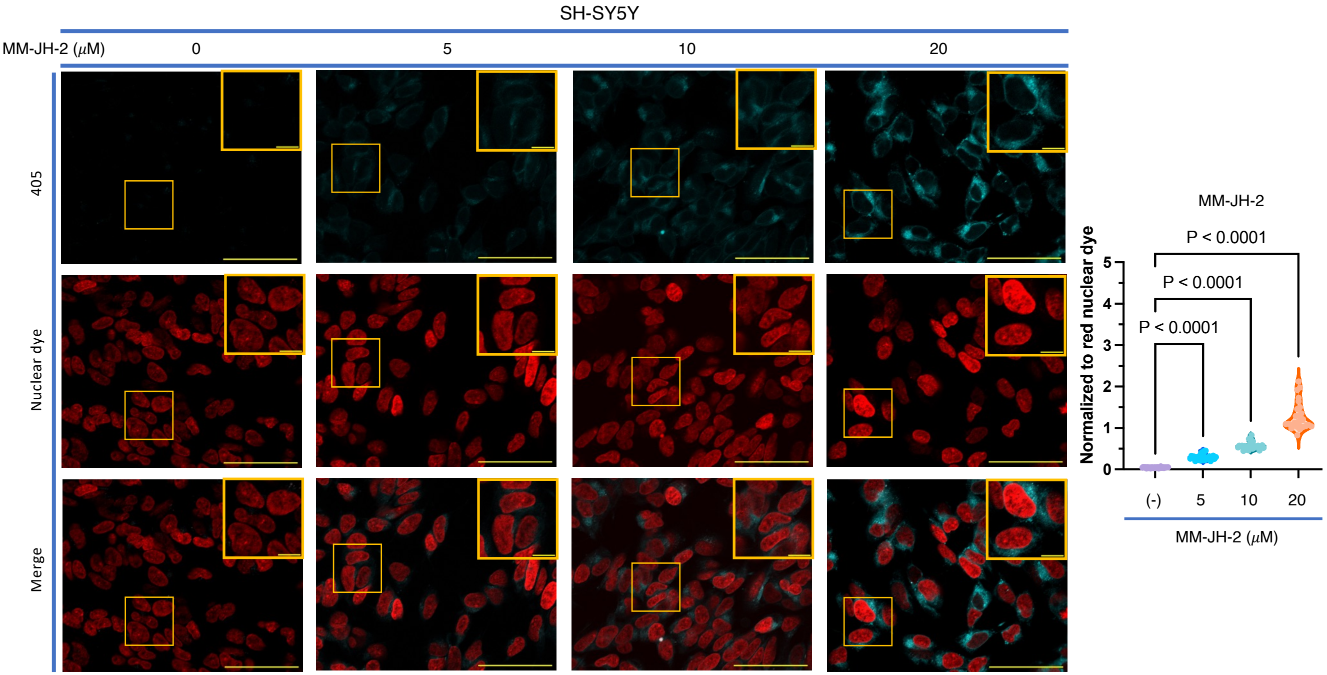


**Supplementary Figure 3**. Concentration dependent incorporation of MM-JH-2 into SH-SY5Y neuroblastoma cells. MM-JH-2 is incorporated into SH-SY5Y cells in a concentration dependent manner, showing detectable labeling from 5 to 20 µM treatment. *N* = 5 individual biological replicates, *n* = 26 individual cells chosen for quantification from the confocal images. An ordinary one-way ANOVA test was performed. P-values are shown in the graph and error bars represent the standard deviation centered on the mean. Scale bars are 50 µm and 10 µm for zoomed images. Quantification is shown to the right of the images.

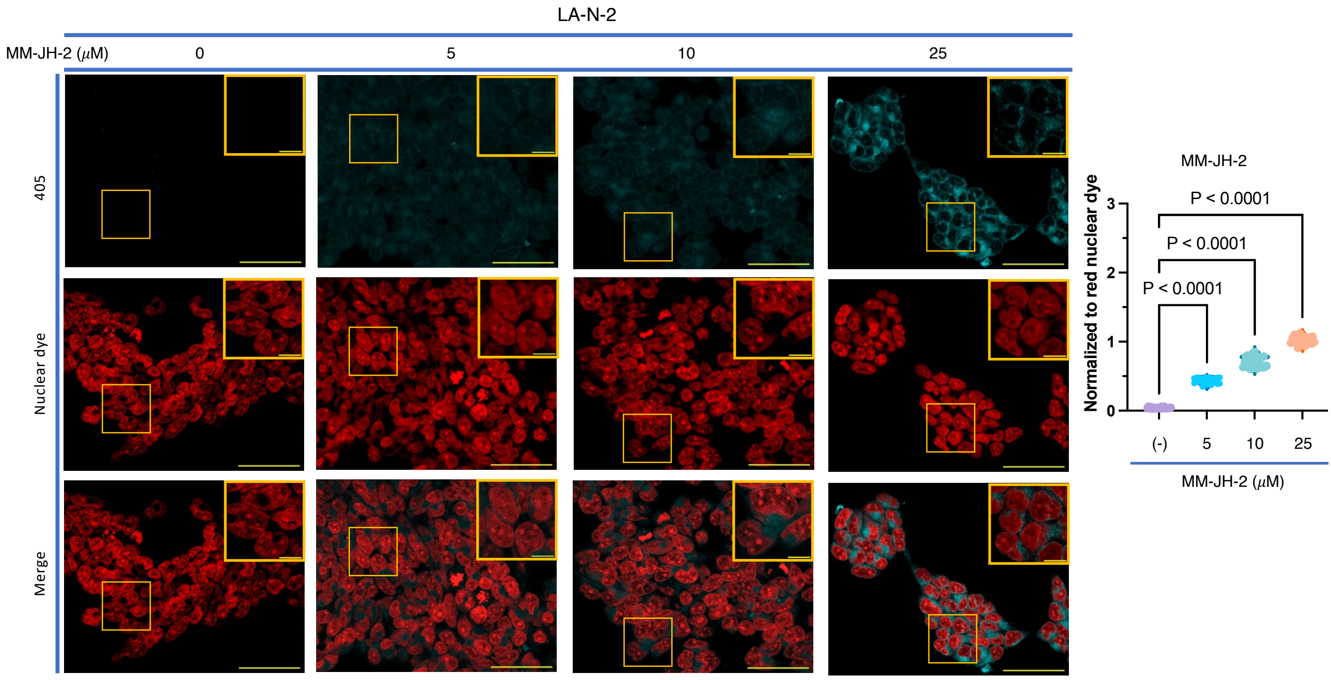


**Supplementary Figure 4.** Concentration dependent incorporation of MM-JH-2 into LA-N-2 neuroblastoma cells. MM-JH-2 is incorporated into LA-N-2 cells in a concentration dependent manner, showing detectable labeling from 5 to 25 µM treatment. $N = 5$ individual biological replicates, $n = 26$ individual cells chosen for quantification from the confocal images. An ordinary one-way ANOVA test was performed. P-values are shown in the graph and error bars represent the standard deviation centered on the mean. Scale bars are 50 µm and 10 µm for zoomed images. Quantification is shown to the right of the images.

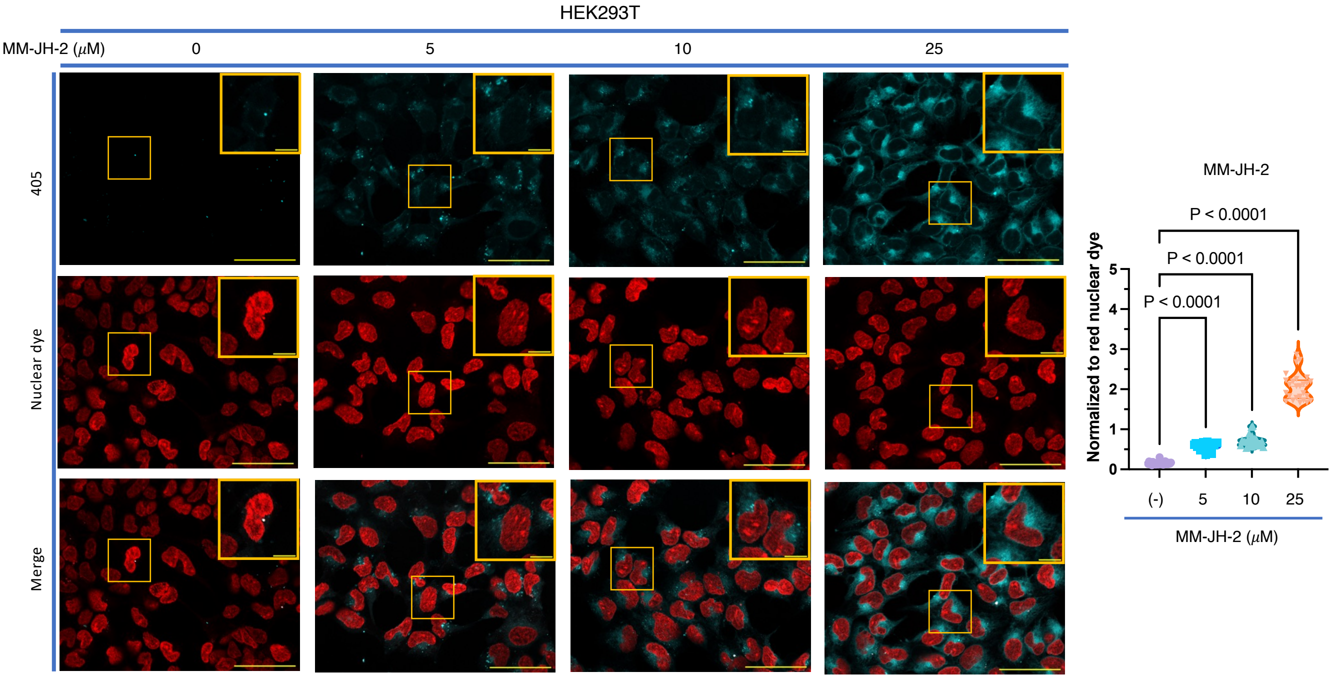


**Supplementary Figure 5.** Concentration dependent incorporation of MM-JH-2 into HEK293T epithelial cells. MM-JH-2 is incorporated into HEK293T cells in a concentration dependent manner, showing detectable labeling from 5 to 25 μM treatment. *N* = 5 individual biological replicates, *n* = 26 individual cells chosen for quantification from the confocal images. An ordinary one-way ANOVA test was performed. P-values are shown in the graph and error bars represent the standard deviation centered on the mean. Scale bars are 50 μm and 10 μm for zoomed images. Quantification is shown to the right of the images.

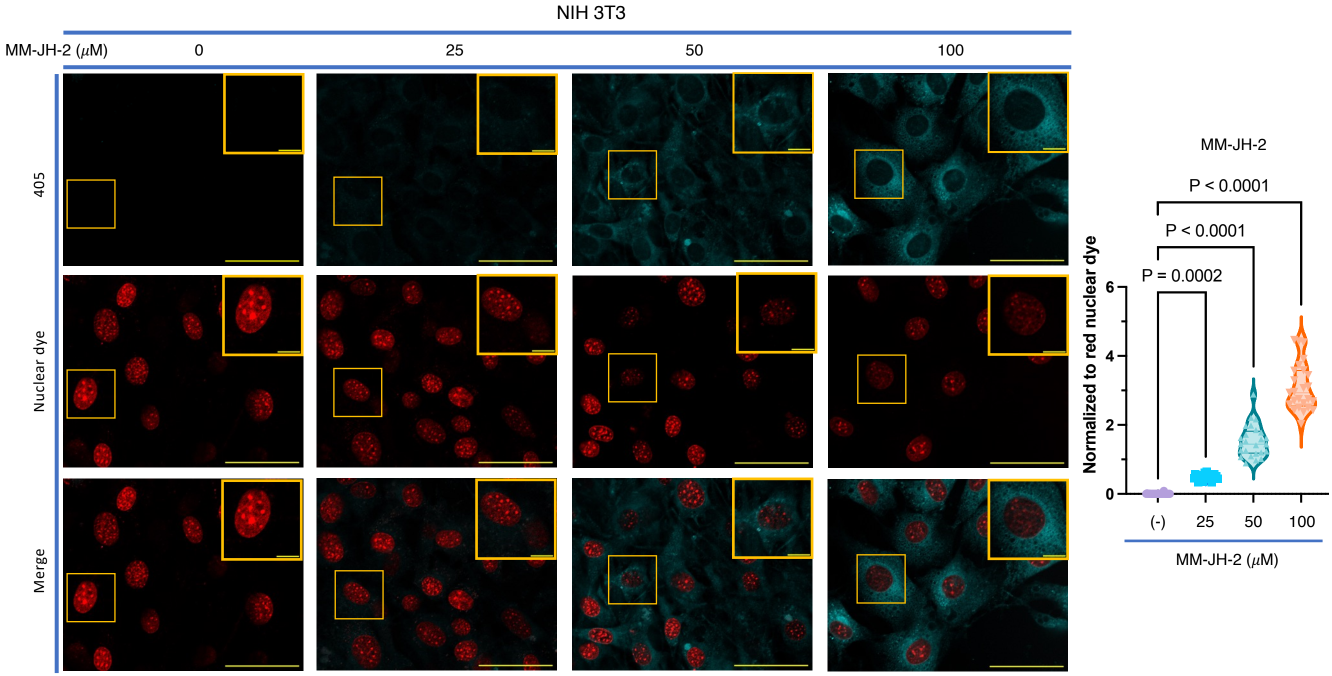


**Supplementary Figure 6**. Concentration dependent incorporation of MM-JH-2 into NIH 3T3 epithelial cells. MM-JH-2 incorporates into NIH 3T3 cells in a concentration dependent manner showing detectable labeling from 50 to 100 μM treatment. N = 5 individual biological replicates, n = 25 individual cells chosen for quantification from the confocal images. An ordinary one-way ANOVA test was performed. P-values are shown in the graph, and the error bars represent the standard deviation with mean as center. Scale bars are 50 μm and 10 μm for zoomed images. Quantification is shown to the right of the images.

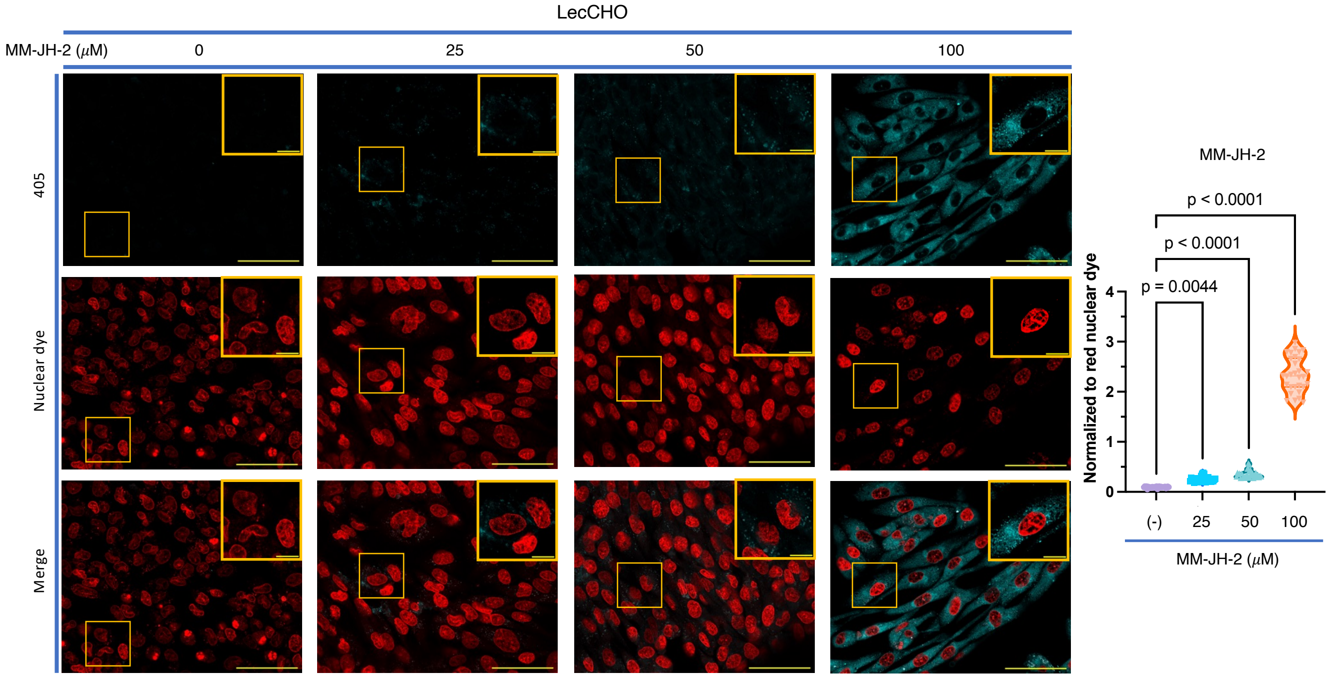


**Supplementary Figure 7.** Concentration dependent incorporation of MM-JH-2 into GnT-I mutated LecCHO epithelial cells. MM-JH-2 is incorporated into LecCHO cells in a concentration dependent manner, showing detectable labeling from 50 to 100 μM treatment. *N* = 5 individual biological replicates, *n* = 30 individual cells chosen for quantification from the confocal images. An ordinary one-way ANOVA test was performed. P-values are shown in the graph and error bars represent the standard deviation centered on the mean. Scale bars are 50 μm and 10 μm for zoomed images. Quantification is shown to the right of the images.

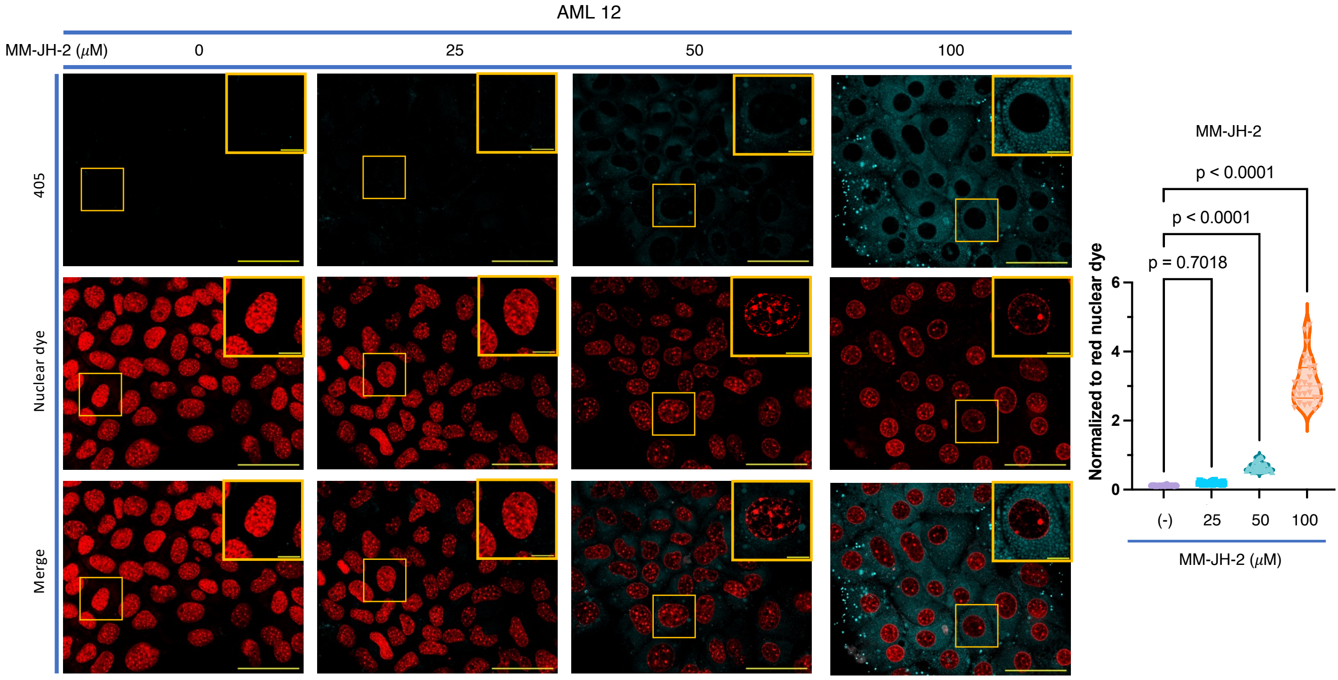


**Supplementary Figure 8**. Concentration dependent incorporation of MM-JH-2 into AML12 mouse hepatocytic cells. MM-JH-2 is incorporated into AML12 cells in a concentration dependent manner, showing detectable labeling from 50 to 100 μM treatment. $N = 5$ individual biological replicates, $n = 30$ individual cells chosen for quantification from the confocal images. An ordinary one-way ANOVA test was performed. P-values are shown in the graph and error bars represent the standard deviation centered on the mean. Scale bars are 50 μm and 10 μm for zoomed images. Quantification is shown to the right of the images.

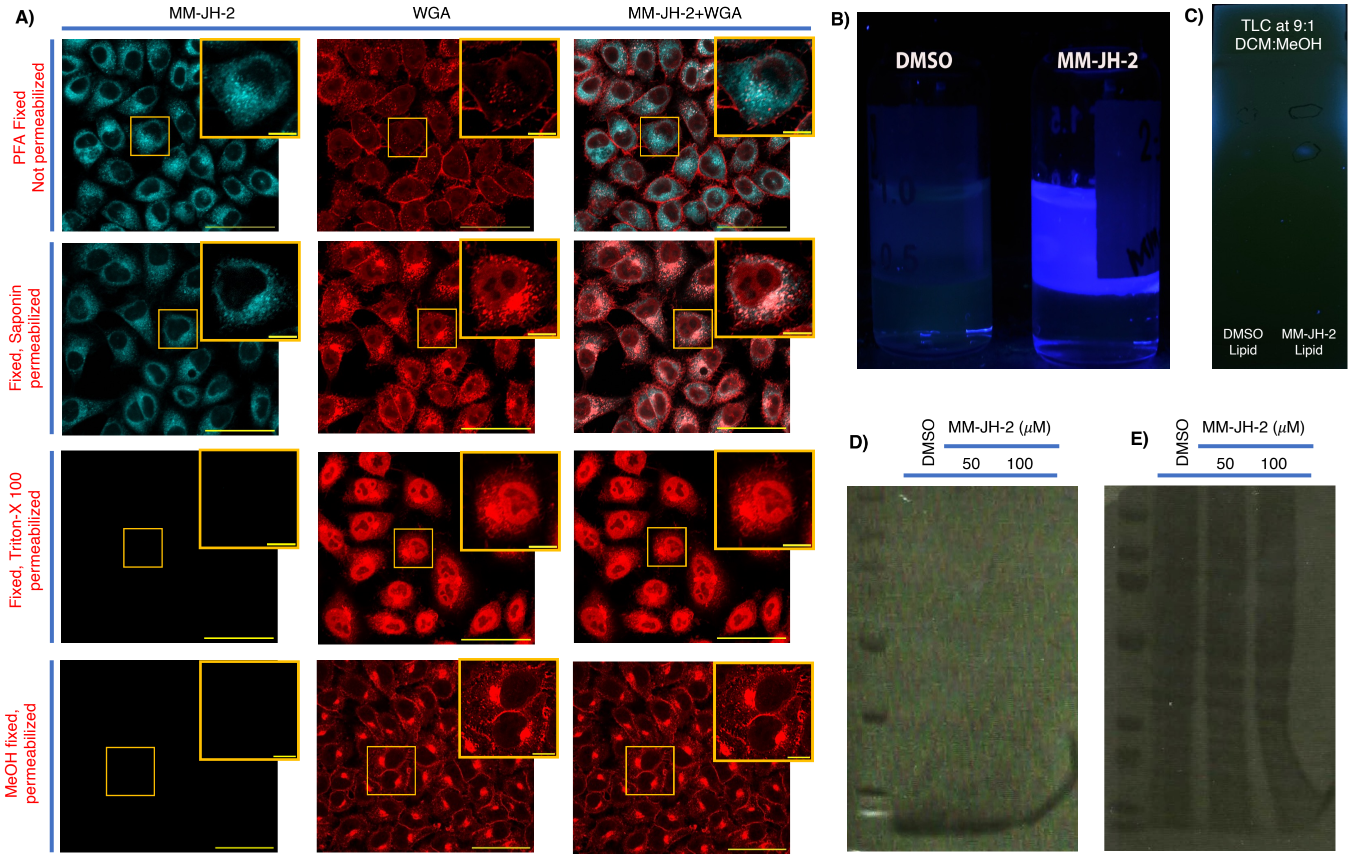


**Supplementary Figure 9.** MM-JH-2 selectively labels lipids in cultured HeLa cells. (A) Effect of permeabilization reagents on MM-JH-2 labeling. The cholesterol-dissolving agent saponin has no effect on MM-JH-2 labeling, but the detergent Triton X-100 and cold methanol completely wash out MM-JH-2 labeled species. *N* = 4 individual biological replicates. Scale bars are 50 µm and 10 µm for zoomed images. (B) Lipid extracts from DMSO and MM-JH-2 treated HeLa cells under UV light showing blue fluorescence in the MM-JH-2 treated extract. *N* = 5 individual biological replicates. (C) TLC analysis of lipid extracts from DMSO and MM-JH-2 treated HeLa cells under UV light. (D) HeLa cells were treated with the indicated concentrations of MM-JH-2 for 72 hours and analyzed by in-get fluorescence scanning. (E) Coomassie blue staining demonstrated equal protein loading. *N* = 3 individual biological replicates.

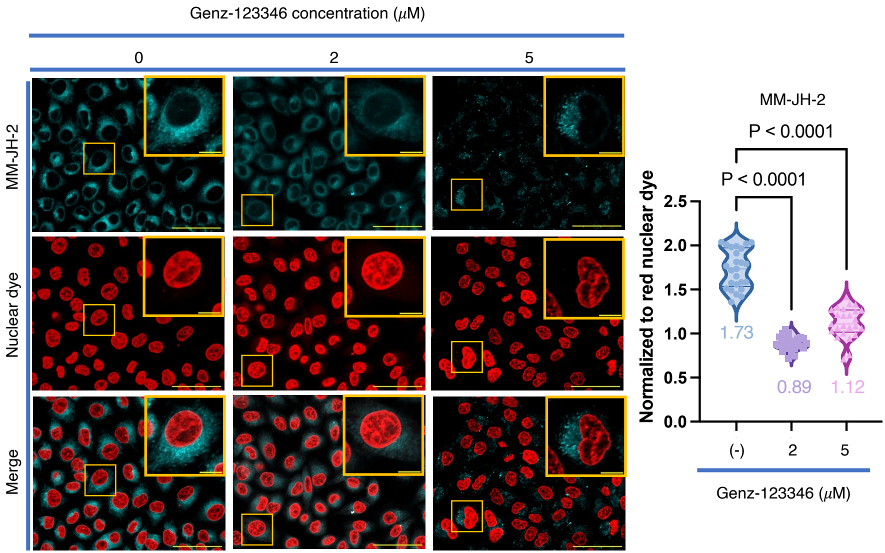


**Supplementary Figure 10.** Glucosylceramide synthase inhibitor Genz-123346 reduces MM-JH-2 labeling in HeLa cells. *N* = 5 individual biological replicates, *n* = 25 individual cells chosen for quantification from the confocal images. An ordinary one-way ANOVA test was performed. P-values are shown in the graph and error bars represent the standard deviation centered on the mean. Scale bars are 50 μm and 10 μm for zoomed images. Quantification is shown to the right of the images.

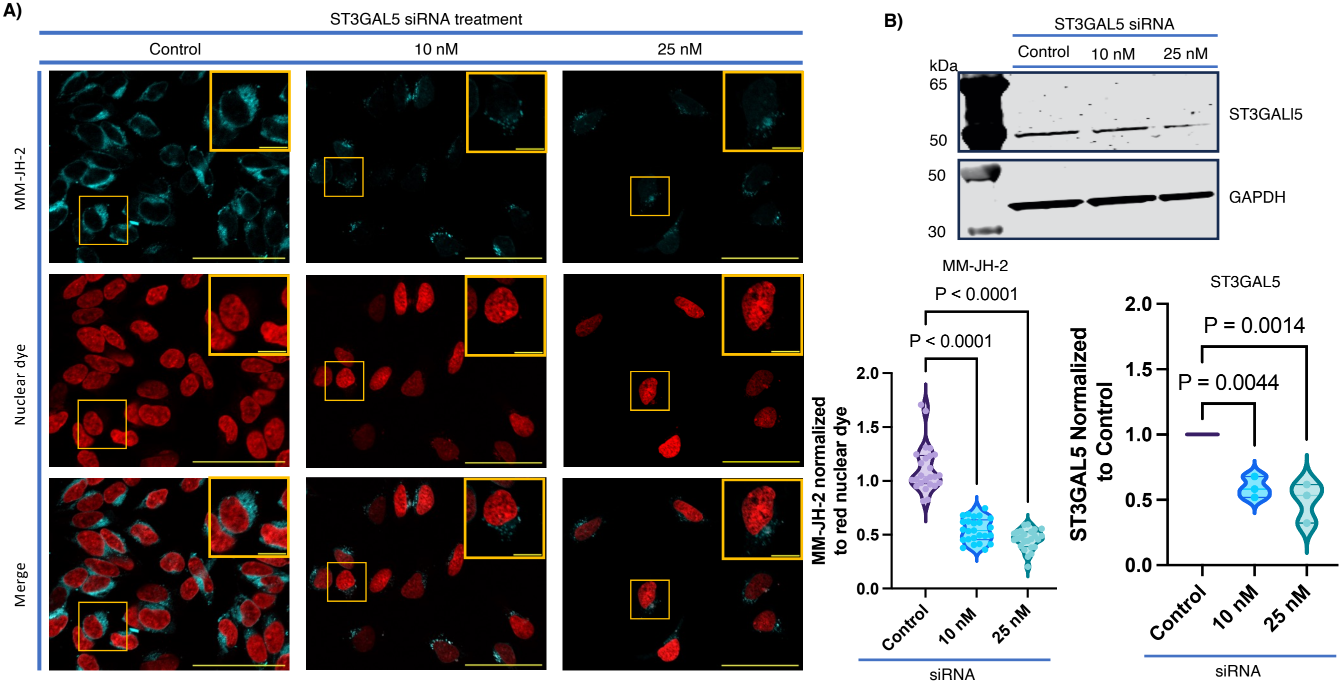


**Supplementary Figure 11.** *ST3GAL5* siRNA knockdown reduces MM-JH-2 labeling in SH-SY5Y cells (A) Confocal fluorescence images of SH-SY5Y cells showing that ST3GAL5 knockdown reduces MM-JH-2 labeling. *N* = 3 individual biological replicates, *n* = 25 individual cells chosen for quantification from the images. An ordinary one-way ANOVA test was performed. P-values are shown in the graph and error bars represent the standard deviation centered on the mean. Scale bars are 50 μm and 10 μm for zoomed images. Quantification is shown to the right of the images. (B) Western blots show ~50% reduction in *ST3GAL5* levels in SH-SY5Y cells upon treatment with siRNA against *ST3GAL5*. *N* = 3 individual biological replicates. An ordinary one-way ANOVA test was performed. P-values are shown in the graph and error bars represent the standard deviation centered on the mean. Quantification is shown at the lower right corner of the images.

A)

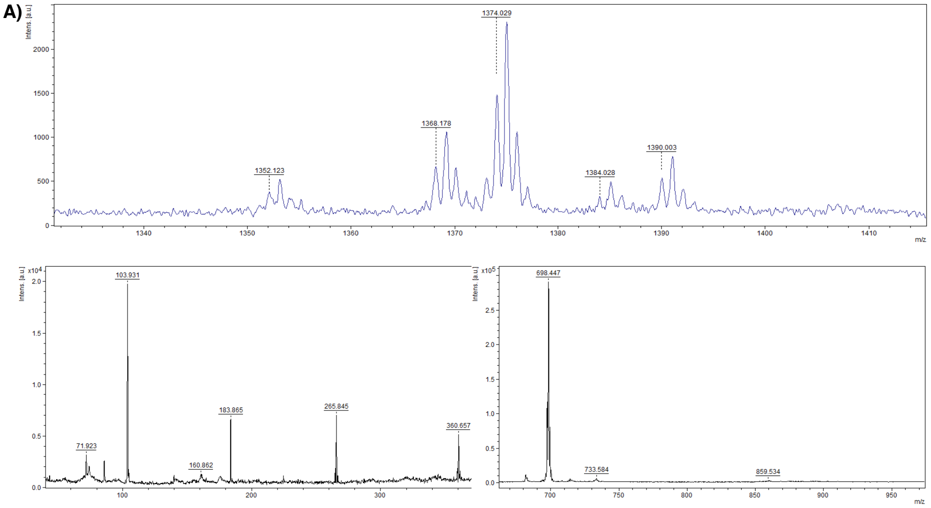



B)

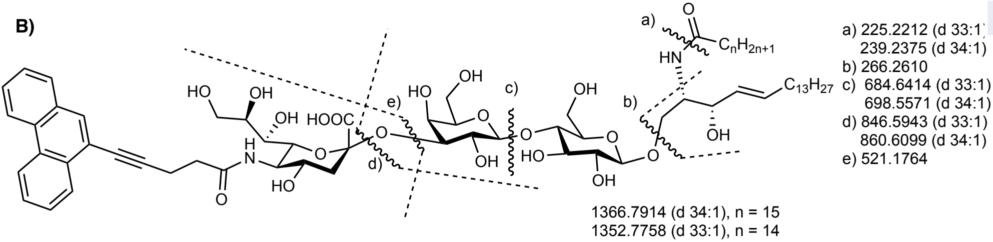



C)

| Peak | Assignment |
|---|---|
| 1391.104 | GM3-MM-JH-2 (d 33:1) + K |
| 1385.112 | GM3-MM-JH-2 (d 34:1) + $H_2O$ |
| 1375.107 | GM3-MM-JH-2 (d 33:1) + Na |
| 1368.868 | GM3-MM-JH-2 (d 34:1) + H |
| 1353.102 | GM3-MM-JH-2 (d 33:1) + H |
| 875.603 | Gal-Glc-Cer (d 33:1) + $H_2O$ |
| 859.542 | Gal-Glc-Cer (d 34:1) |
| 698.466 | Glc-Cer (d 34:1) |
| 543.873 | Cer (d 34:1) + Na |
| 521.631 | Neu5-Alk-phenanthrene |
| 517.829 | Cer (d 33:1) |
| 360.663 | Gal-Glc |
| 265.832 | Cer C18 |
| 224.754 | Cer C15; Cer (d 33:1) |

D)

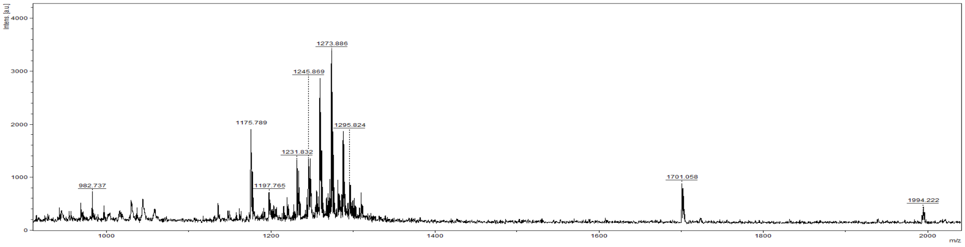



**Supplementary Figure 12.** MALDI mass spectrometric analysis identifies MM-JH-2 modified GM3 gangliosides in lipid extracts. (A) MALDI mass spectrum of a lipid extract from MM-JH-2 treated NIH 3T3 cells. (B) Chemical structure and possible fragmenting position of GM3-MM-JH-2. (C) Peak assignments for possible MALDI mass fragmentations. (D) MALDI mass spectra of a lipid extract from DMSO treated NIH 3T3 cells. *N* = 3 individual biological replicates.

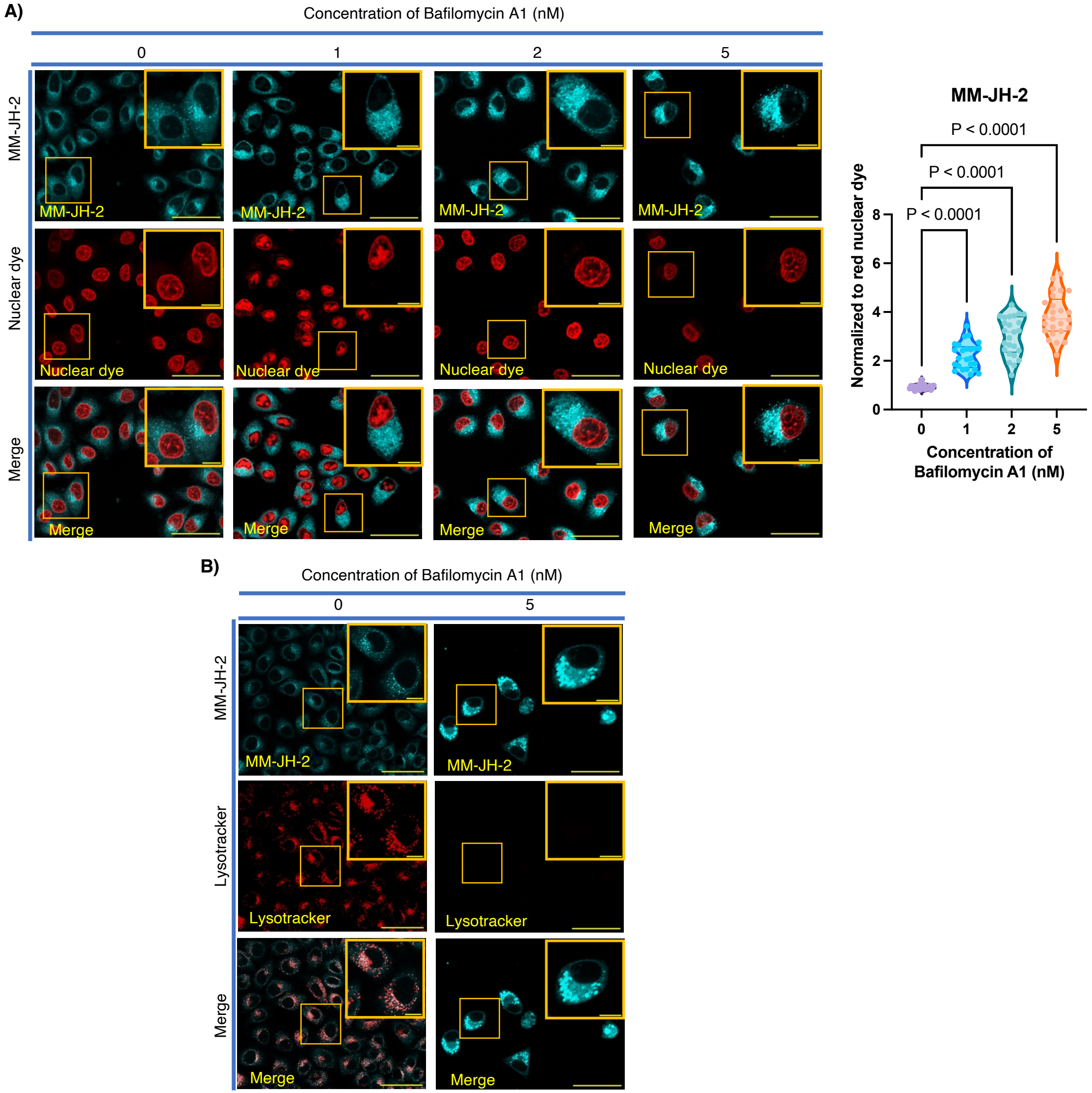


**Supplementary Figure 13.** MM-JH-2 localizes to lysosomes and late endosomes for catabolism. (A) The ATPase inhibitor bafilomycin A1 increased the fluorescence intensity of MM-JH-2 (blue) in lysosomes. $N = 5$ individual biological replicates, $n = 25$ individual cells chosen for quantification from the confocal images. An ordinary one-way ANOVA test was performed. P-values are shown in the graph and error bars represent the standard deviation centered on the mean. Scale bars are 50 μm and 10 μm for zoomed images. Quantification is shown to the right of the images. (B) Bafilomycin A1 treatment resulted in a reduction of acidity in lysosomes and late endosomes. $N = 3$ individual biological replicates. Scale bars are 50 μm and 10 μm for zoomed images.

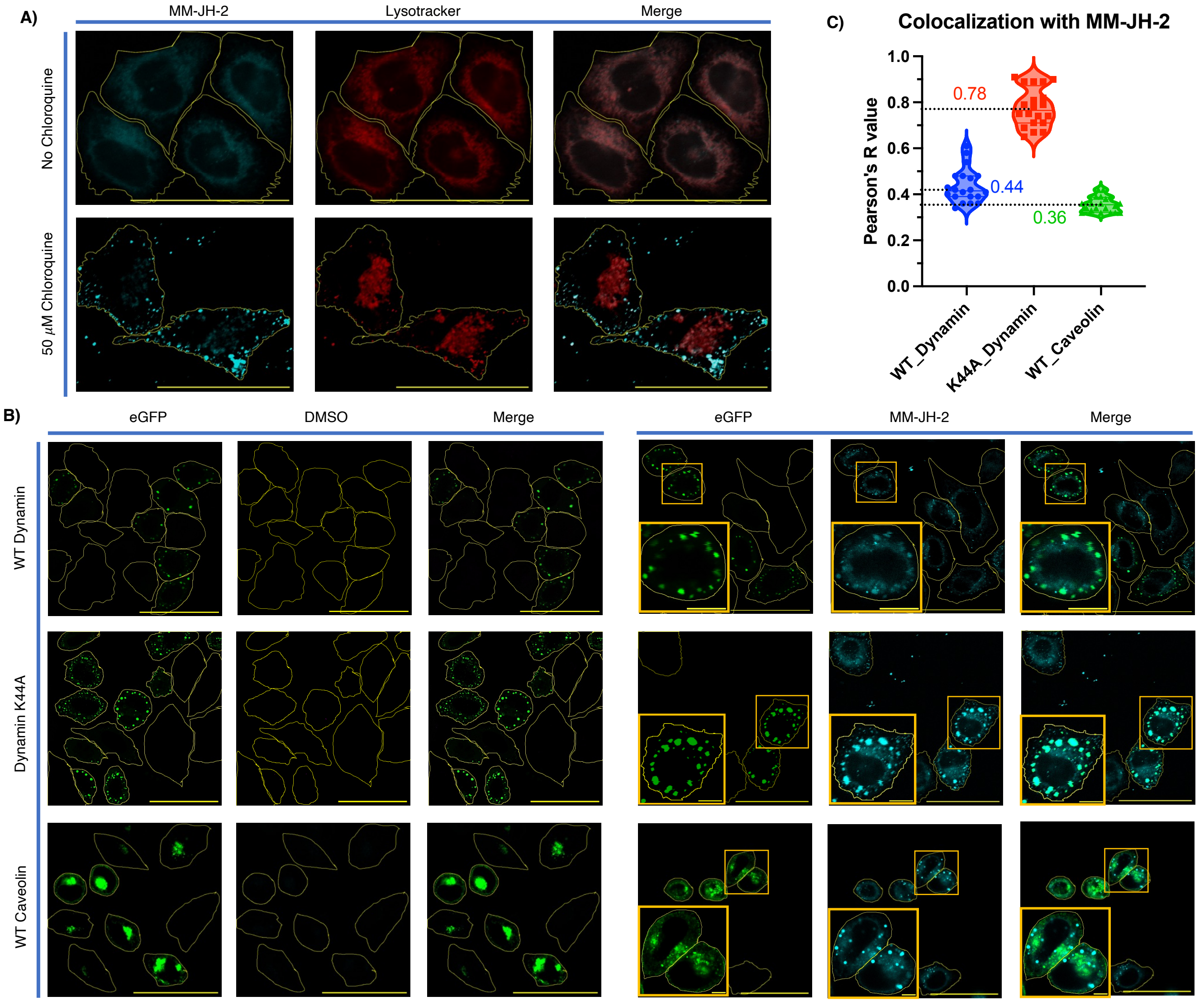


**Supplementary Figure 14.** MM-JH-2 endocytosis depends on both dynamin and caveolin. (A) Confocal images showing that chloroquine treatment blocks MM-JH-2 endocytosis in HeLa cells and results in localization of MM-JH-2 to the plasma membrane. $N = 3$ individual biological replicates. The scale bar is 50 μm. (B) Confocal fluorescence images of HeLa cells transfected with eGFP-dynamin (WT and the K44A mutant) and WT caveolin. $N = 3$ individual biological replicates. Scale bars are 50 μm and 10 μm for zoomed images. (C) Colocalization of MM-JH-2 (blue) with eGFP signals. Colocalization was quantified by mean Pearson's R value. $N = 4$ individual biological replicates, $n = 20$ individual cells chosen for Pearson's R value analysis from the confocal images. Error bars represent the standard deviation centered on the mean.

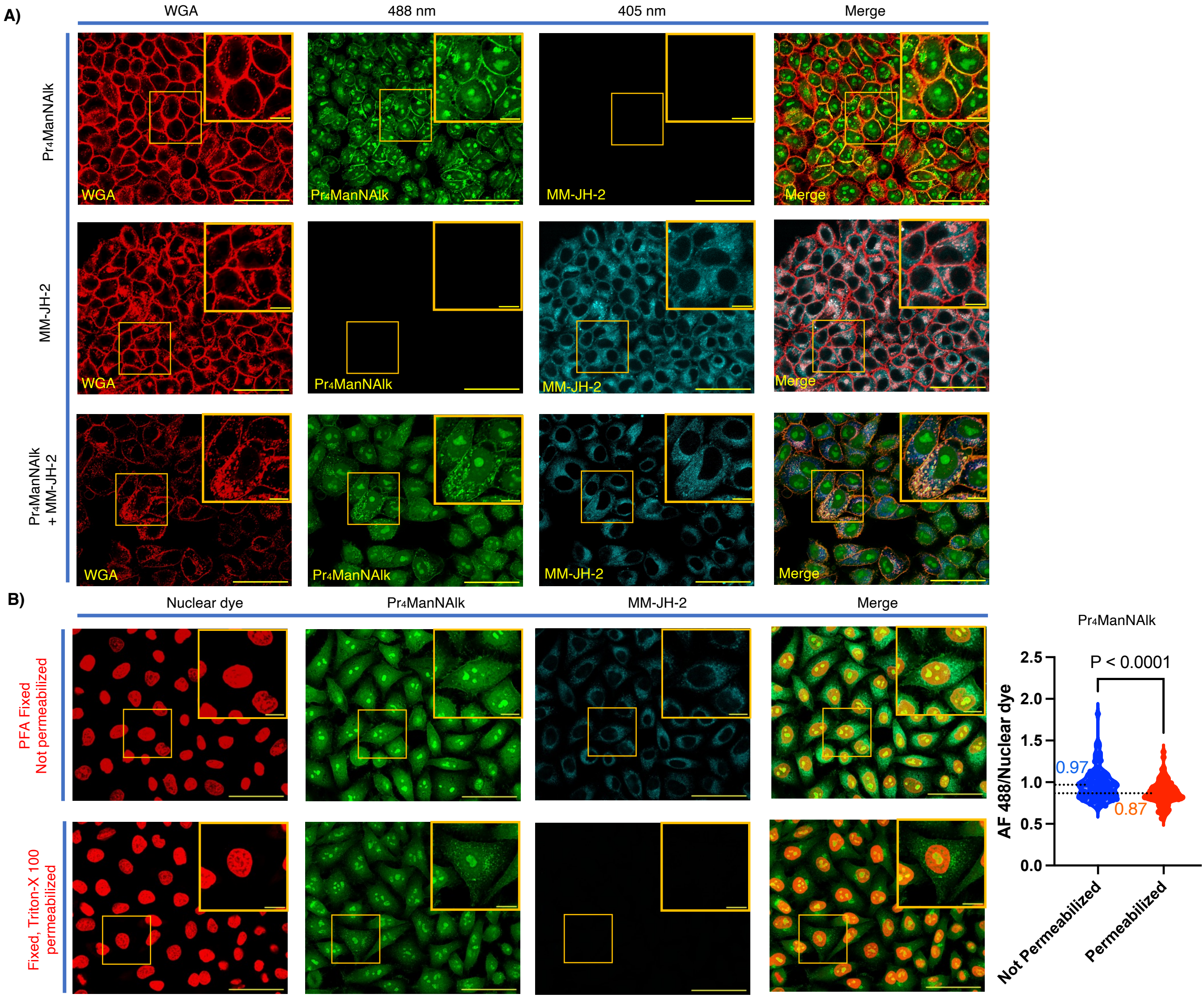


**Supplementary Figure 15.** Confocal imaging of HeLa cells treated with MM-JH-2 and $Pr_4ManNAlk$. (A) These two MOEs label HeLa cells differentially and only superimpose in intracellular structures, but not on the PM nor in the nucleus. MM-JH-2 labeling was present in intracellular structures but not on the cell surface. $Pr_4ManNAlk$ was distributed predominantly on the PM with some nonspecific labeling of the nucleolus. $N = 5$ individual biological replicates. (B) Approximately 10% of the total $Pr_4ManNAlk$ signal is associated with glycolipid labeling. $N = 5$ individual biological replicates, $n = 100$ individual cells chosen for quantification from the confocal images. A two-sided unpaired $t$-test was performed. Quantification is shown to the right of the images. P-values are shown in the graph and error bars represent the standard deviation centered on the mean. Scale bars are 50 μm and 10 μm for zoomed images.

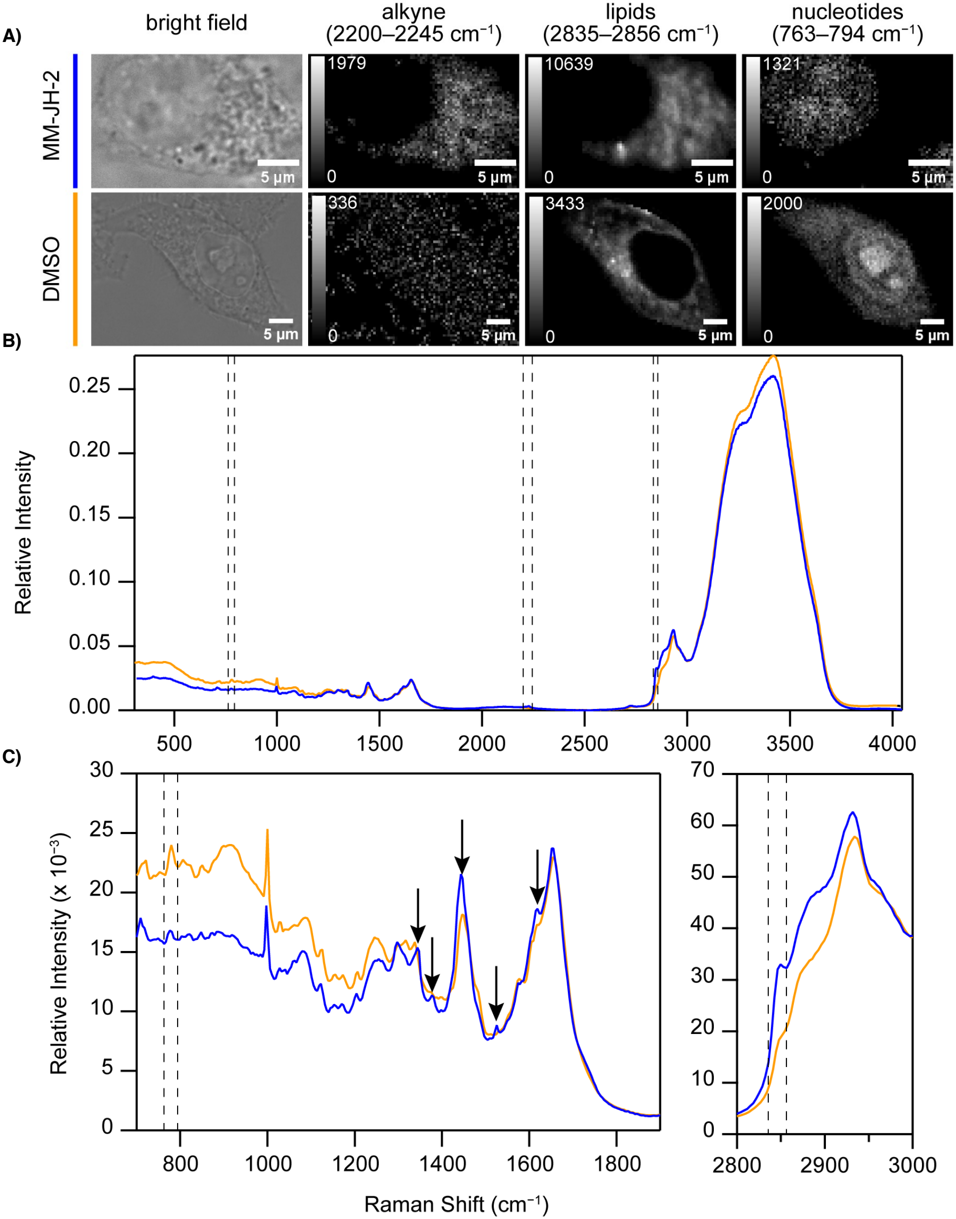


**Supplementary Figure 16.** Raman spectral imaging of HeLa cells treated with MM-JH-2. (A) Raman maps of the cells used to generate the averaged spectra shown in (B) and (C) as well as Fig. 4B. (B) Full spectral window of the averaged whole-cell Raman spectra shown in Fig. 4B showing the spectral regions used to generate maps of nucleotides, MM-JH-2, and lipids (dashed lines). (C) Detail of the fingerprint (left) and C–H stretching (right) regions of the Raman spectrum. Additional spectral features of MM-JH-2 in the fingerprint region are indicated by arrows.

A)

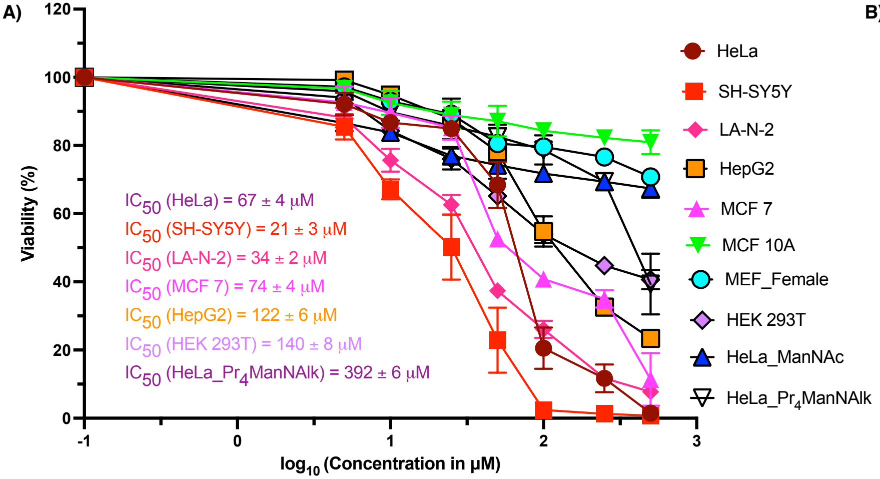


B)

| Cell Line | IC50 (µM) | Concentrations (µM) used in the study |
|---|---|---|
| HeLa | 67 ± 4 | 10-50 |
| SH-SY 5Y | 21 ± 3 | 5-20 |
| LA-N-2 | 34 ± 2 | 5-25 |
| HepG2 | 122 ± 6 | -- |
| MCF 7 | 74 ± 8 | 5-25 |
| MCF 10A | ND | 25-100 |
| MEF (Female) | ND | -- |
| HEK 293T | 140 ± 8 | 5-25 |

**Supplementary Figure 17.** MM-JH-2 selectively eradicates cancer cells over nonmalignant cells. (A) MTT assays of various cell lines treated with MM-JH-2 (5 to 500 µM) show that only malignant cells (HeLa, MCF7, SH-SY5Y, LA-N-2, HepG2) are eradicated by the treatment, whereas no considerable cytotoxicity was observed for nonmalignant cell lines (MEF, MCF10A) up to 500 µM treatment. ND indicates no detection up to 500 µM MM-JH-2 treatment. *N* = 6 individual biological replicates. Error bars represent the standard deviation centered on the mean. (B) Table of $IC_{50}$ values for MM-JH-2 in the cell lines used in this study.

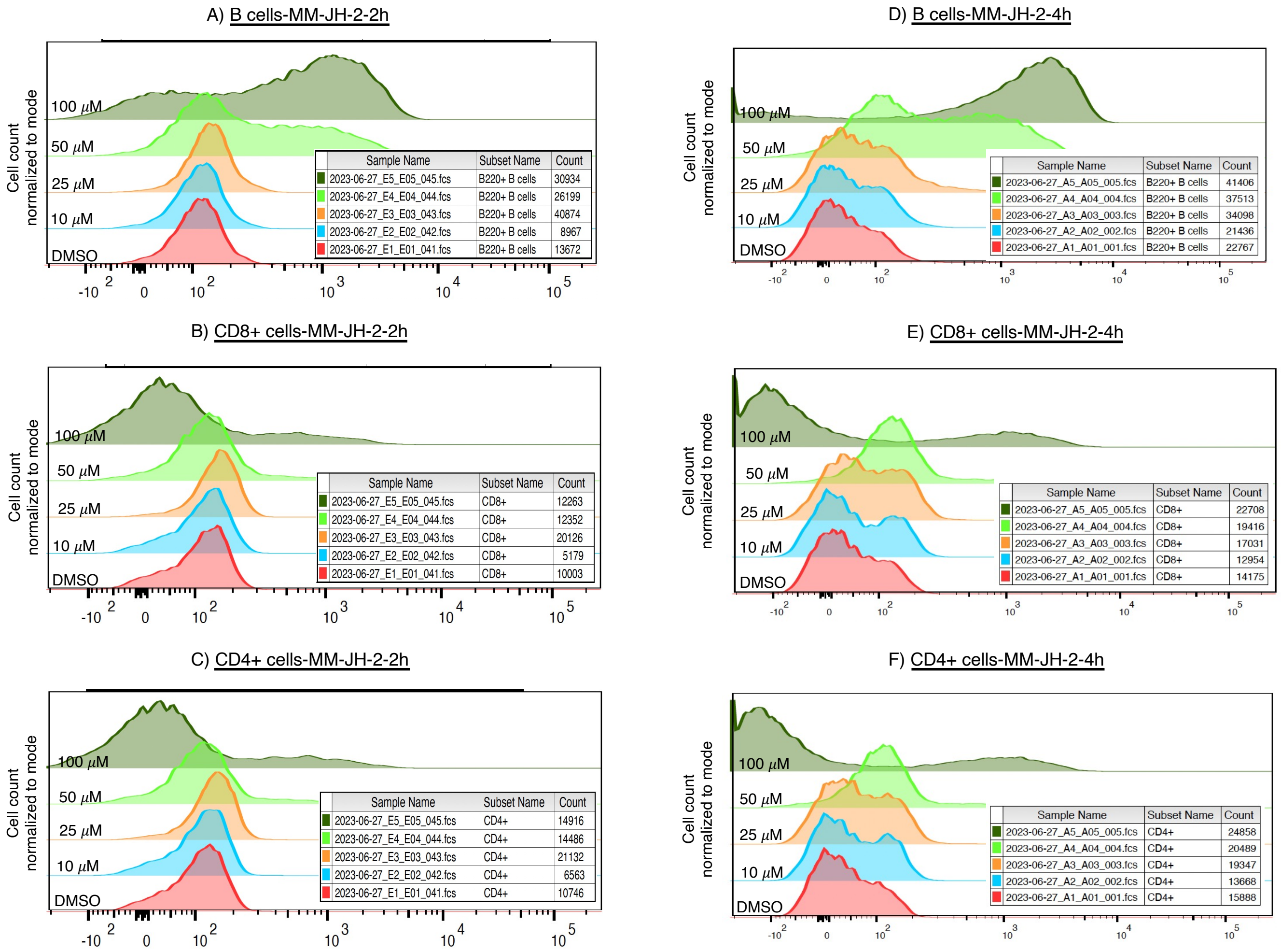


**Supplementary Figure 18.** MM-JH-2 can selectively label B cells over T cells in a coculture of splenocytes. Histograms showing the change in (A) B220$^+$-cell, (B) $CD_8^+$-cell, and (C) $CD_4^+$-cell populations after treatment with MM-JH-2 for 2 hours; (D) B220$^+$-cell, (E) $CD_8^+$-cell, and (F) $CD_4^+$-cell populations after treatment with MM-JH-2 for 4 hours. *N* = 6 individual biological replicates.

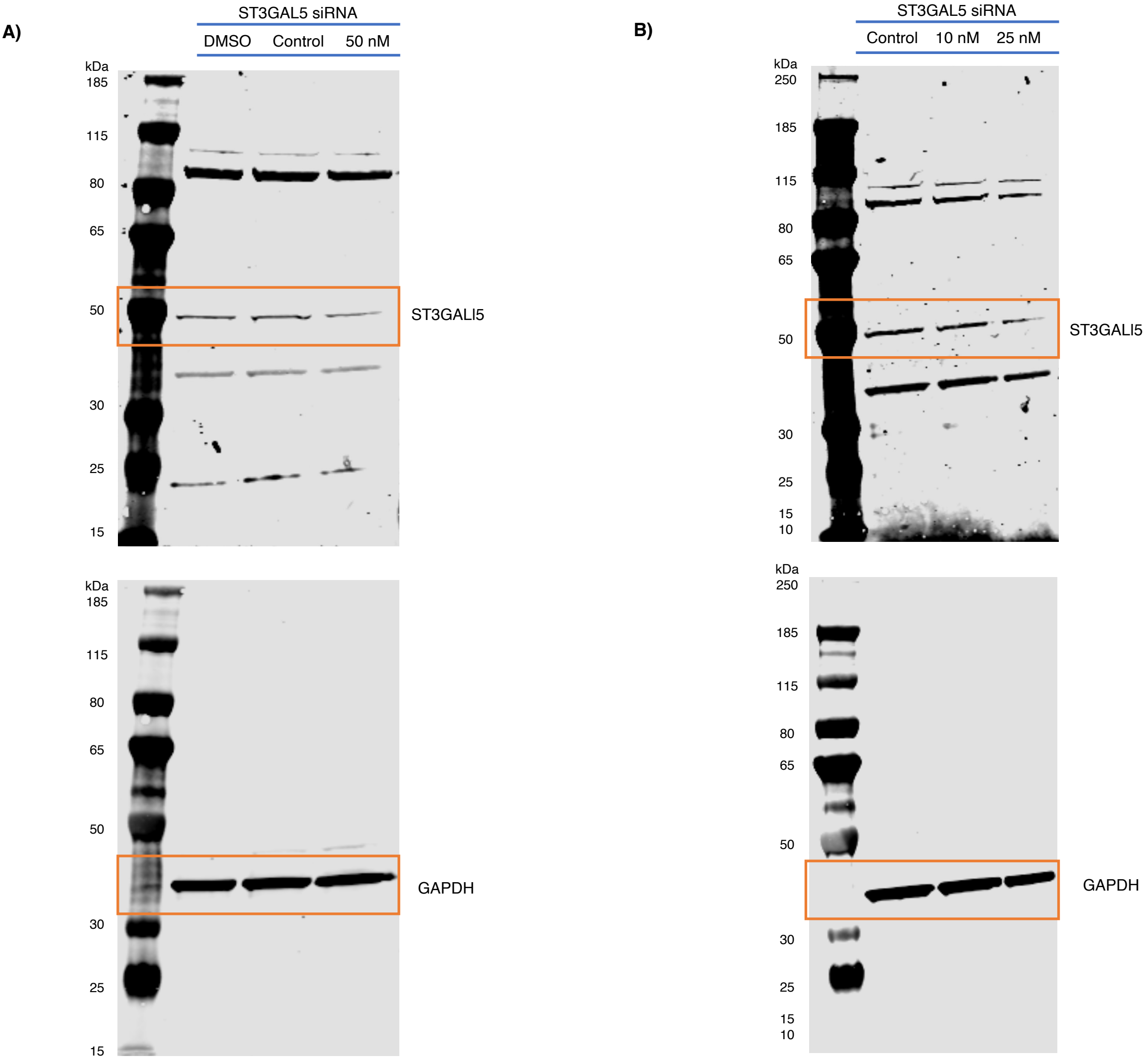


**Supplementary Figure 19.** Full uncropped western blots showing *ST3GAL5* siRNA knockdown reduces *ST3GAL5* labeling in (A) HeLa cells, and (B) SH-SY5Y cells.
