# Supplementary material for: A dual fluorescent-Raman bioorthogonal probe for specific biosynthetic labeling of intracellular gangliosides
Source: Commun Chem. 2025 Oct 3;8:293. doi: 10.1038/s42004-025-01685-x (PMC12494992; doi:10.1038/s42004-025-01685-x)
Supplement: Supplementary file 3 — Description of Additional Supplementary Files [file 42004_2025_1685_MOESM3_ESM.pdf]

Description of Additional Supplementary Files

File name: Supplementary Data 1

Description: Numerical source data files for Main Figures

File name: Supplementary Data 2

Description: Numerical source data files for Supplementary Information Figures
