# Supplementary material for: A dual fluorescent-Raman bioorthogonal probe for specific biosynthetic labeling of intracellular gangliosides
Source: Commun Chem. 2025 Oct 3;8:293. doi: 10.1038/s42004-025-01685-x (PMC12494992; doi:10.1038/s42004-025-01685-x)
Supplement: Supplementary file 6 — Hanover_Mukherjee nr-reporting-summary [file 42004_2025_1685_MOESM6_ESM.pdf]

nature portfolio

Corresponding author(s): John A. Hanover

Last updated by author(s): Aug 22, 2025

Reporting Summary

Nature Portfolio wishes to improve the reproducibility of the work that we publish. This form provides structure for consistency and transparency in reporting. For further information on Nature Portfolio policies, see our Editorial Policies and the Editorial Policy Checklist.

Statistics

For all statistical analyses, confirm that the following items are present in the figure legend, table legend, main text, or Methods section.

n/a	Confirmed	
☐	☒	The exact sample size (*n*) for each experimental group/condition, given as a discrete number and unit of measurement
☐	☒	A statement on whether measurements were taken from distinct samples or whether the same sample was measured repeatedly
☐	☒	The statistical test(s) used AND whether they are one- or two-sided<br>*Only common tests should be described solely by name; describe more complex techniques in the Methods section.*
☐	☒	A description of all covariates tested
☐	☒	A description of any assumptions or corrections, such as tests of normality and adjustment for multiple comparisons
☐	☒	A full description of the statistical parameters including central tendency (e.g. means) or other basic estimates (e.g. regression coefficient) AND variation (e.g. standard deviation) or associated estimates of uncertainty (e.g. confidence intervals)
☐	☒	For null hypothesis testing, the test statistic (e.g. *F*, *t*, *r*) with confidence intervals, effect sizes, degrees of freedom and *P* value noted<br>*Give P values as exact values whenever suitable.*
☒	☐	For Bayesian analysis, information on the choice of priors and Markov chain Monte Carlo settings
☒	☐	For hierarchical and complex designs, identification of the appropriate level for tests and full reporting of outcomes
☐	☒	Estimates of effect sizes (e.g. Cohen's *d*, Pearson's *r*), indicating how they were calculated

Our web collection on statistics for biologists contains articles on many of the points above.

Software and code

Policy information about availability of computer code

Data collection	NMR: Bruker Avance Spectrometer using TopSpin (4.1.1)<br>BCA: Microsoft Excel<br>Western Blot: Image studio (5.2.5)<br>Immunofluorescence: Zeiss LSM700 (63X water lens) using Zen 2.3 SP1 FP3 (14.0.0.0)<br>MALDI Mass: AutoFlex III (Bruker)<br>Raman imaging: Infinity 3S-1URM CCD (Lumenera)<br>Flow cytometry: LSRFortessa flow cytometer using BD FACS Diva (version 8.0; BD Biosciences)
Data analysis	NMR: TopSpin (4.1.1)<br>BCA: Microsoft Excel<br>Quantification: GraphPad Prism (10)<br>Western Blot analysis: Image studio (5.2.5)<br>Immunofluorescence: ImageJ (FiJi 2.14.0/1.54f)<br>Raman spectra analysis: LabSpec6 software package (Horiba)<br>Flowcytometry: Flowjo v10.9.0 software package (BD Biosciences)

For manuscripts utilizing custom algorithms or software that are central to the research but not yet described in published literature, software must be made available to editors and reviewers. We strongly encourage code deposition in a community repository (e.g. GitHub). See the Nature Portfolio guidelines for submitting code & software for further information.

Data

Policy information about availability of data

All manuscripts must include a data availability statement. This statement should provide the following information, where applicable:

- Accession codes, unique identifiers, or web links for publicly available datasets
- A description of any restrictions on data availability
- For clinical datasets or third party data, please ensure that the statement adheres to our policy

All data supporting the findings of this study are available within the paper and its Supplementary Information files. Should any raw data files be needed in another format they are available from the corresponding author upon reasonable request. Source data are provided with this paper.

Research involving human participants, their data, or biological material

Policy information about studies with human participants or human data. See also policy information about sex, gender (identity/presentation), and sexual orientation and race, ethnicity and racism.

Reporting on sex and gender	Information about Reporting on sex and gender is not applicable as the research was conducted on cultured eukaryotic cell lines. Reporting of sex and gender is not relevant for the studies conducted with mice.
Reporting on race, ethnicity, or other socially relevant groupings	Information about Reporting on race, ethnicity, or other social relevant groupings are not applicable as the research was conducted on cultured eukaryotic cell lines. Reporting of sex and gender is not relevant for the studies conducted with mice.
Population characteristics	Information about Population characteristics is not applicable as the research was conducted on cultured eukaryotic cell lines.
Recruitment	Information about Recruitment is not applicable as the research was conducted on cultured eukaryotic cell lines.
Ethics oversight	Information about Ethics oversight is not applicable as the research was conducted on cultured eukaryotic cell lines.

Note that full information on the approval of the study protocol must also be provided in the manuscript.

Field-specific reporting

Please select the one below that is the best fit for your research. If you are not sure, read the appropriate sections before making your selection.

☒ Life sciences ☐ Behavioural & social sciences ☐ Ecological, evolutionary & environmental sciences

For a reference copy of the document with all sections, see nature.com/documents/nr-reporting-summary-flat.pdf

Life sciences study design

All studies must disclose on these points even when the disclosure is negative.

Sample size	For independent experiments, at least three replicates are conducted. For quantification of confocal data, 10 cells were quantified for each experiments.
Data exclusions	No data were excluded.
Replication	Yes, at least three replicates and the exact numbers of experimental replicates are noted in the figure legends.
Randomization	Independent experiments like immunoblotting or immunocytochemistry or flowcytometry or mass spectrometric analysis can not be randomized because the experiments were done on eukaryotic cell lines which are genetically identical and did not involve assignment of individuals or treatment groups.
Blinding	Independent experiments like immunoblotting or immunocytochemistry or flowcytometry can not be blinded because the experiments did not involve allocation of different samples, organisms, or participants into experimental groups.

Reporting for specific materials, systems and methods

We require information from authors about some types of materials, experimental systems and methods used in many studies. Here, indicate whether each material, system or method listed is relevant to your study. If you are not sure if a list item applies to your research, read the appropriate section before selecting a response.

Materials & experimental systems

n/a	Involved in the study	
☐	☒	Antibodies
☐	☒	Eukaryotic cell lines
☒	☐	Palaeontology and archaeology
☐	☒	Animals and other organisms
☒	☐	Clinical data
☒	☐	Dual use research of concern
☒	☐	Plants

Methods

n/a	Involved in the study	
☒	☐	ChIP-seq
☐	☒	Flow cytometry
☒	☐	MRI-based neuroimaging

Antibodies

Antibodies used

The primary antibodies (1:1000 dilution for Western Blot and 1:100 dilution for Immunofluorescence):
Mouse anti GAPDH (Abcam ab8245; 1:1000)
Rabbit anti GAPDH (Abcam, ab18078; 1:1000)
Rabbit anti LAMP1 (Cell Signaling Technologies, 9091S; 1:1000)
Rabbit anti ST3GAL5 (Novus Biologicals, NBP2-20492; 1:1000)
Allophycocyanin (APC)-CD4+ (Thermo Fisher Scientific, 17-0042-82; 1:200)
Fluorescein isothiocyanate (FTIC)-CD8+ (Thermo Fisher Scientific, 11-0081-85; 1:200)
Phycoerythrin (PE)-B220+ (Thermo Fisher Scientific, 12-0452-82; 1:200).
The secondary antibodies: (1:10000 dilution for Western Blot and 1:500 dilution for Immunofluorescence)
IRDyeâ 680 RD goat anti-mouse IgG (H+L) (Li-COR, 926-68070; 1:10000)
IRDyeâ 680 RD goat anti-rabbit IgG (H+L) (Li-COR, 926-68071; 1:10000)
IRDyeâ 800 CW goat anti-mouse IgG (H+L) (Li-COR, 926-32210; 1:10000)
IRDyeâ 800 CW goat anti-rabbit IgG (H+L) (Li-COR, 926-32211; 1:10000)
AlexaFluor 568 goat anti-rabbit (Invitrogen, A11011; 1:500)

Validation

All the antibodies listed above have been validated by the manufacturers (see website links below)
Mouse anti GAPDH (Abcam ab8245; https://www.abcam.com/products/primary-antibodies/gapdh-antibody-6c5-loading-control-ab8245.html)
Rabbit anti GAPDH (Abcam, ab18078; https://www.abcam.com/products/primary-antibodies/gapdh-antibody-loading-control-ab9485.html)
Rabbit anti LAMP1 (Cell Signaling Technologies, 9091S; https://www.cellsignal.com/products/primary-antibodies/lamp1-d2d11-xp-174-rabbit-mab/9091)
Rabbit anti ST3GAL5 (Novus Biologicals, NBP2-20492; https://www.novusbio.com/products/st3gal5-antibody_nbp2-20492)
Allophycocyanin (APC)-CD4+ (Thermo Fisher Scientific, 17-0042-82; https://www.thermofisher.com/antibody/product/CD4-Antibody-clone-RM4-5-Monoclonal/17-0042-82)
Fluorescein isothiocyanate (FTIC)-CD8+ (Thermo Fisher Scientific, 11-0081-85; https://www.thermofisher.com/antibody/product/CD8a-Antibody-clone-53-6-7-Monoclonal/11-0081-85)
Phycoerythrin (PE)-B220+ (Thermo Fisher Scientific, 12-0452-82;https://www.thermofisher.com/antibody/product/CD45R-B220-Antibody-clone-RA3-6B2-Monoclonal/12-0452-82)
IRDyeâ 680 RD goat anti-mouse IgG (H+L) (Li-COR, 926-68070; https://www.licor.com/bio/reagents/irdye-680rd-goat-anti-mouse-igg-secondary-antibody)
IRDyeâ 680 RD goat anti-rabbit IgG (H+L) (Li-COR, 926-68071; https://www.licor.com/bio/reagents/irdye-680rd-goat-anti-rabbit-igg-secondary-antibody)
IRDyeâ 800 CW goat anti-mouse IgG (H+L) (Li-COR, 926-32210; https://www.licor.com/bio/reagents/irdye-800cw-goat-anti-mouse-igg-secondary-antibody)
IRDyeâ 800 CW goat anti-rabbit IgG (H+L) (Li-COR, 926-32211; https://www.licor.com/bio/reagents/irdye-800cw-goat-anti-rabbit-igg-secondary-antibody)
AlexaFluor 568 goat anti-rabbit (Invitrogen, A11011; https://www.thermofisher.com/antibody/product/Goat-anti-Rabbit-IgG-H-L-Cross-Adsorbed-Secondary-Antibody-Polyclonal/A-11011)

Eukaryotic cell lines

Policy information about cell lines and Sex and Gender in Research

Cell line source(s)

HeLa (ATCC, CRM-CCL-2), Mouse Embryonic Fibroblasts (MEFs), MCF7 (ATCC, HBT-22), MCF 10A (ATCC, CRL-10317), HEK 293T (ATCC, CRL-3216), NIH 3T3 (ATCC, CRL-1658), HepG2 (ATCC, HB-8065), LA-N-2 (Sigma, 06041202-1VL), SH-SY5Y (ATCC, CRL-2266), LecCHO (CRL-1735), AML12 (CRL -2254)

Authentication

Cell lines were not authenticated and used as supplied by the vendor

Mycoplasma contamination

Cell lines were tested for mycoplasma contaminations and they tested negative for mycoplasma contaminations

Commonly misidentified lines
(See ICLAC register)

No misidentified cell lines were used

Animals and other research organisms

Policy information about studies involving animals; ARRIVE guidelines recommended for reporting animal research, and Sex and Gender in Research

Laboratory animals	C57Bl/6J-Mgea5tm2Jah/cre mice (3-Male, 3-Female) were analyzed between 10-12 weeks of age
Wild animals	Study did not involve wild animals
Reporting on sex	Study did not involve sex-based analysis as B and T cell collected from mice spleen are equivalent irrespective of their sex.
Field-collected samples	Study did not involve samples collected from the field
Ethics oversight	The animals were maintained according to the animal protocol #K023-LCBB-22 approved by the NIDDK Animal Care and Use Committee, National Institutes of Health, USA.

Note that full information on the approval of the study protocol must also be provided in the manuscript.

Plants

Seed stocks	Study did not involve plant stock
Novel plant genotypes	Study did not involve plant stock
Authentication	Study did not involve plant stock

Flow Cytometry

Plots

Confirm that:

☒ The axis labels state the marker and fluorochrome used (e.g. CD4-FITC).

☒ The axis scales are clearly visible. Include numbers along axes only for bottom left plot of group (a 'group' is an analysis of identical markers).

☒ All plots are contour plots with outliers or pseudocolor plots.

☒ A numerical value for number of cells or percentage (with statistics) is provided.

Methodology

Sample preparation	Total spleen was isolated from mice and tissue was disrupted mechanically in the presence of complete RMPI media and passed through a 70 um cell strainer washing with the complete media. Cell numbers are counted after ACK lysis using a Nexcelon Cellometer and 1.5*10^3 cells were plated per well of a 96-well plate and incubated with different concentrations of MM-JH-2 (25, 50 and 100 uM) for different times (2 h and 4 h) and correspondingly with DMSO as negative control. Thereafter washed thrice with flow cytometry staining buffer (Thermo Fisher scientific, 00-4222-26) and block with 100 uL of flow cytometry blocking buffer (Anti Mo CD16/CD32 blocking Thermo Fisher scientific, 14-0161-85, 1:100 dilution in flow cytometry staining buffer) for 15 min at 4 oC. For five colored flow cytometry analysis, cells were incubated with APC-CD4+ (Thermo Fisher Scientific, 17-0042-82), FTIC-CD8+ (Thermo Fisher Scientific, 11-0081-85), PE-B220+ (Thermo Fisher Scientific, 12-0452-82) for 30 min at room temperature (antibodies were used in 1:200 dilution in flow cytometry staining buffer). To minimize the levels of nonspecific bindings, cells were washed three times in flow cytometry staining buffer and finally resuspended in 150 uL of flow cytometry staining buffer followed by addition of 5 uL PI (Thermo Fisher Scientific, 00-6990-42) solution for live/dead differentiation.
Instrument	BD LSRFortessa flow cytometer equipped with 355nm, 407nm, 488nm, 532nm and 640nm laser lines and high throughput sampler system (BD Biosciences).
Software	Data were recorded using BD FACS Diva software v 8.0 and recorded fcs files were analyzed using Flowjo v10 software (both from BD Biosciences)
Cell population abundance	Immune cell subsets such as T cells, B cells, and remaining primary cells from mouse spleen
Gating strategy	Debris free single live cells (as no Propidium Iodide uptake) were identified using cell physical parameters (FSC/SSC). Live single cells were further gated for B cells as B220+, CD4+ T cells as B220- CD8-CD4+ cells, CD8+ T cells as B220-CD4-CD8+

cells, whereas remaining cells containing heterogeneous cell populations as B220-CD4-CD8- live single cells. Uptake of fluorescent probe as correlative of MM-JH-2 was excited by UV laser line and read using 450-50 nm band pass filter. fluorescence minus one (FMO) control containing cell surface antibodies but not probe (MM-JH-2) was used to define presence of dye and as correlative for uptake of MM-JH-2. Data is presented as % positive subsets for probe as well as mean fluorescence intensity.

☒ Tick this box to confirm that a figure exemplifying the gating strategy is provided in the Supplementary Information.
